# Building a Genomics-Informed Nursing Workforce: Recommendations for Oncology Nursing Practice and Beyond

**DOI:** 10.3390/curroncol32010014

**Published:** 2024-12-27

**Authors:** Jacqueline Limoges, Rebecca Puddester, Andrea Gretchev, Patrick Chiu, Kathy Calzone, Kathleen Leslie, April Pike, Nicole Letourneau

**Affiliations:** 1 Faculty of Health Disciplines, Athabasca University, Athabasca, AB T9S 3A3, Canada; agretchev@athabascau.ca (A.G.); kleslie@athabascau.ca (K.L.); 2 Faculty of Nursing, Memorial University Newfoundland, St. John’s, NL A1B3V6, Canada; rjp823@mun.ca (R.P.); aprilpike@mun.ca (A.P.); 3 Faculty of Nursing, University of Alberta, Edmonton, AB T6G 1C9, Canada; pakcheon@ualberta.ca; 4 National Cancer Institute, Genetics Branch, Center for Cancer Research, Bethesda, MD 20892, USA; calzonek@mail.nih.gov; 5 Faculty of Nursing, University of Calgary, Calgary, AB T2N 1N4, Canada; nicole.letourneau@ucalgary.ca

**Keywords:** nursing, genomics, leadership, policy, professional practice

## Abstract

Background: Genomics is a foundational element of precision health and can be used to identify inherited cancers, cancer related risks, therapeutic decisions, and to address health disparities. However, there are structural barriers across the cancer care continuum, including an underprepared nursing workforce, long wait times for service, and inadequate policy infrastructure that limit equitable access to the benefits of genomic discoveries. These barriers have persisted for decades, yet they are modifiable. Two distinct waves of efforts to integrate genomics into nursing practice are analyzed. Drawing on research and observations during these waves, this discussion paper explores additional approaches to accelerate workforce development and health system transformation. Results: Three recommendations for a third wave of efforts to integrate genomics are explored. (1) Collaborate across the domains of nursing practice, professions, and sectors to reset priorities in response to emerging evidence, (2) Education in leadership, policy and practice for rapid scale-up of workforce and health system transformation, and (3) Create a research framework that generates evidence to guide nursing practice. Conclusions: Preparing nurses to lead and practice at the forefront of innovation requires concerted efforts by nurses in all five domains of practice and can optimize health outcomes. Leveraging nursing as a global profession with new strategies can advance genomics-informed nursing.

## 1. Introduction

Nurse leaders called for initiatives to address genetic advancements in the 1960s [1], recognizing that the growing availability and applicability of genomics would transform healthcare. Advancements in genomics are now enhancing the safety and quality of cancer prevention, detection, treatment, and symptom management [2,3,4] while reducing the overall cancer burden [5,6]. Genomics is a foundational element of precision health and can be used to identify inherited diseases, the accuracy of risk predictions, therapeutic decisions, and to address health disparities [4,7]. However, structural barriers including an underprepared health workforce, long wait times for service, and inadequate policy infrastructure are limiting equitable access and distribution of these benefits across the cancer care continuum [8,9,10,11,12,13,14,15]. To address these structural barriers, all members of the healthcare workforce must have genomic literacy [16] and integrate genomics into interprofessional practice. While there have been tremendous efforts across generations of nurses [17,18,19] and growing urgency to develop nursing policy, educational initiatives, and research, the Canadian nursing workforce remains largely unprepared for the genomics era [20,21,22,23]. This challenge is not unique to Canada, as nurses in other countries also report low levels of genomic literacy and difficulties engaging with genomic services, pointing to a global issue with nursing workforce development and health system transformation [18,19,24]. Specifically in oncology, studies in Turkey [25] and Australia [26] have revealed similar patterns where oncology nurses see the relevance of genomics yet require education to meet patient needs. This is problematic, particularly in cancer care, which now commonly includes genomics [4,27]. Therefore, to enhance nurses’ contributions to genomics-informed health system transformation, strategies to accelerate workforce development are critical.

In this discussion paper, we review historical and contemporary waves of efforts to integrate genomics into nursing practice and draw on our program of research and observations as nurses leading genomics integration initiatives in Canada [20,22,23,28,29,30,31,32]. The recent cross-jurisdictional policy analysis [30] provided an opportunity to engage in a focused analysis of genomics-informed initiatives in the United States (US) and the United Kingdom (UK), which are two jurisdictions that have made significant progress. Drawing on past and current efforts in nursing workforce development, we offer three recommendations for the next wave of strategic action. These recommendations are relevant to nurses in all five domains of practice: clinical care, administration, policy, research, and education. 

Note: Genetics studies individual genes, their inheritance, and their role in disease phenotypes. Genomics examines all genes in the genome and their interactions with each other and the environment, offering a more comprehensive view of health and disease. While “genetics” was once used broadly, “genomics” is now preferred when not specifically addressing the study of single-gene disorders. This paper will use “genomics” except when referring to historical research that used “genetics” to maintain alignment with original sources.

## 2. The Historical Trajectory: Canadian Nursing and Genomics Wave 1.0 (c. 2004–2006)

In response to the landmark sequencing of the human genome in 2003 and its forecasted impact on health, federally funded research initiatives were used to launch the first wave of activities to prepare the Canadian nursing workforce. Early research included needs and knowledge assessments, and environmental scans [33,34,35,36]. This work led to five recommendations to prepare the nursing workforce for genomics integration (many of which are still relevant today) [36]. Recommended strategies included defining competencies to guide education and practice, providing education, raising awareness about the relevance of genetics, lobbying for the development of the genetic nurse, and the creation of networks of Canadian nurses to foster mentorship opportunities and research. At the time, these efforts aligned with other countries, including the US and UK [30]. Additionally, in 2005, Bottorff et al. [34] explored the roles of ‘genetic nurses’ in Canada—many of whom were providing genetic counselling. At that time, more than a third of these nurses had been practicing in the field for over 20 years, demonstrating lengthy nursing involvement with genetic services [34]. However, this early momentum in nursing was lost. In 1991, 23.3% of official job titles in Canadian genetic counselling centers contained the word ‘nurse’; by 2006, this number fell to 2.7% as retiring nurses were replaced with other professionals holding master’s degrees in genetic counselling [37].

During this period, when genetic nurses were being phased out and replaced with genetic counsellors (GCs), our research revealed several gaps. We found no evidence of support for interprofessional education or collaboration between nurses and GCs; no opportunities for credentialing nurses with genomics expertise or including nurses in providing genomic services, and no evidence that any of the five recommendations by Bottorff [36] were implemented [22,34]. Therefore, as Bottorff et al. [35] predicted, by 2009, the already limited pool of genomic nurses in Canada was depleted. While no published documents trace how these decisions were made, we learned through conversations that nursing organizations turned their focus to other topics and did not see the relevance of genomics to nursing, halting all prior momentum for genomics-informed nursing. This lack of investment and prioritization in sustaining or growing the genomics nursing workforce and supporting interprofessional collaboration occurred in the early 2000s, despite rapidly growing genomics discoveries [38], increasing numbers of nursing publications [18,39], billions of dollars being invested worldwide by governments and private sectors for genomic research [40] and when the benefits of genomics were increasingly evident [41].

## 3. Contemporary Canadian Nursing and Genomics Wave 2.0 (c. 2020–2024)

The decisions made during the first wave of genomics integration into clinical care continue to impact Canadian nurses’ abilities to provide the genomics-informed health services that Canadians now expect. There are insufficient numbers of expert nurses to champion entry-to-practice and continuing nursing education, nursing research and clinical leadership, or to build and implement vital policy infrastructure to support nursing practice [20,21,22,29]. For example, when the Canadian Nursing and Genomics (CNG) [42] steering group began engagement and outreach in 2020, we struggled to recruit a handful of Canadian nurses with clinical genomics expertise to drive action. Previous research highlights the hindrance to innovation, safety and workforce development when there is no policy response to incorporate new evidence into practice [43] and the crucial role of leaders and champions in supporting genomics integration [44,45]. As a result, examining the infrastructure and clinical leadership related to nursing and genomics became an important area of exploration in the second wave of Canadian efforts and research.

The CNG initiative and program of research in Canada began in 2020 [42]. An engagement framework was created and initially used to identify nurses’ six priorities for action related to nursing and genomics [29]. These six priorities guided seven research projects that were completed to provide evidence to support the integration of genomics into nursing education, policy, and practice. This research demonstrates how the lack of infrastructure supporting professional practice continues to impede Canadian nurses’ ability to provide safe, compassionate, ethical, evidence-informed, and accountable genomics-informed care [20,22,23,28,29,30,31,32].

We learned that nurses in practice report a rise in genomics-related questions from patients who turn to them as trusted healthcare providers [20,23]. At the same time, nurses report feeling unprepared and unsupported to address genomic patient care needs [20,21,23,46]. This lack of genomic literacy hinders nurses from contributing to the interdisciplinary teams providing genomic services [20]. Nurses describe sitting on the sidelines reluctant to engage, as they grapple with role ambiguity, a lack of interprofessional collaboration and limited education on genomics [20,21,29]. In recent studies, few participants could identify nurse leaders or champions to drive genomics integration and were not aware of where to obtain clinical knowledge to support genomics-informed practices [20,23]. Despite being motivated to increase genomic knowledge, nurses are unsure how to proceed without competency frameworks, education, and policy to guide education or practice [20]. These findings are strikingly similar to the situation in the first wave of Canadian nursing and genomics—20 years ago [33,34,35].

Through comparative policy analysis of genomic nursing policies in the US, UK, and Canada, we learned that the infrastructure and support for nurses unfolded differently in the US and UK, with stronger advancement of nursing and genomics [30]. We found that there were many ideas, interests, institutions and external factors that influenced the integration of genomics into nursing, such as nurses’ values in aligning with evidence and public expectations, strong nursing leadership, policy networks and shifting healthcare landscapes. The Canadian context differed from those two countries and thus has a different state of readiness for the genomic era [20,22,23,28,29,30,31,32].

For example, in the early 1990s, the US was like Canada, with the removal of nurses from genomics roles. However, nurse leaders in the US responded with nursing-specific policy infrastructure for all nurses with graduate degrees, such as the *Essential Genetic and Genomic Competencies for Nurses with Graduate Degrees* [47] and the development of a pathway for genomic nursing credentialing through the American Nurses Credentialing Center [30]. Further, nurses in the US have had genomics competency statements for nearly 20 years, which were recently updated [48]. In the UK, the Association of Genetic Nurses and Counsellors (AGNC) represents nurses and GCs. This enables the AGNC to address professional boundaries between nurses and GCs, guide the scope of practice, and address overlap and distinctions among nurses and GCs [49,50]. The UK addressed professional boundaries by clearly delineating roles, educational requirements, and regulatory responsibilities, advanced by effective leadership, advocacy, and organizational and government support [50,51,52]. Nurses practicing in an advanced capacity receive education to act as leaders and champions of genomics in the US and UK [53,54,55]. These initiatives highlight the positive impact of leadership, policy and concerted strategies on preparing and sustaining the nursing workforce for the genomics era. Analysis of the US and UK context and other Canadian research provides valuable insights on workforce development and recommendations for future strategies.

Our research identified significant gaps in Canadian scholarship to support evidence-based practice. In a recent scoping review, almost all evidence of nursing strategies to address health disparities associated with genomics was produced by US scholars, and none was produced by Canadian scholars [31]. As the Canadian health system is different from the US, with unique equity concerns such as our history with colonization, nurses require context-specific evidence to guide culturally safe integration strategies. Furthermore, while challenges with the evaluation of education interventions are not unique to Canada [56,57], only one Canadian study [28] examined an educational intervention aimed at developing nursing leadership competencies in genomics. Additionally, nursing faculty have reported low levels of genomic literacy and barriers to including genomics in undergraduate nursing education in Canada [58,59]. As nurses require evidence and education to guide implementation strategies to address the theory-practice gap in genomics [60,61], these findings provide clarity on what is required to support genomics-informed nursing.

In nursing and genomics wave 2.0, we benefitted from growing scholarship and acceptance of implementation science on how to support genomic integration [62,63,64,65]. Knowledge synthesis projects revealed how deficit-based narratives on nurses’ low genomic literacy and the primarily descriptive studies limited efforts to galvanize and sustain the integration of genomics. These studies also highlighted the importance of focusing on intervention studies [18,56], policy advocacy, and policy development [20,22,30,66] as initiatives that could accelerate the integration of genomics. Having learned about the impact of oncology nurses’ perceived relevance to engagement in genomics, in the early stages of nursing and genomics 2.0, we pivoted to strategically focus on oncology nursing as the relevance of genomic testing enhanced the sense of urgency to implementation efforts [67]. We also benefited from research that established consensus on core competencies for oncology nurses through the synthesis of published literature and consultation with international experts [68].

## 4. New Approaches and Recommendations: Nursing and Genomics 3.0 (c. 2025)

**Figure 1:**
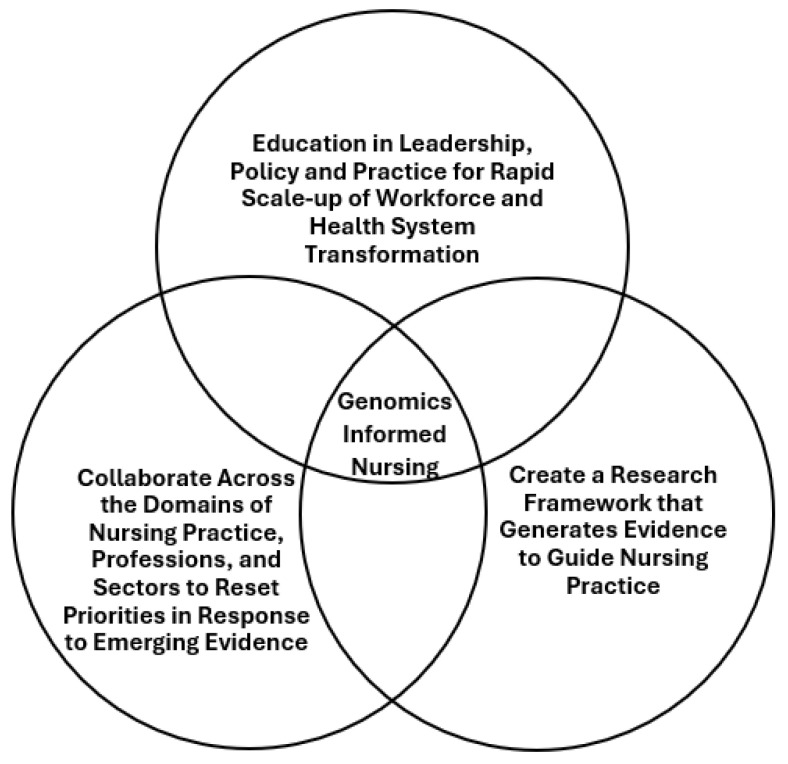
Nursing and Genomics 3.0.

Exploring nursing and genomics 1.0 and 2.0 is not a critique of those waves and decisions. Instead, we described this history to illustrate how decisions made without viable alternatives can leave a lasting impact for generations of nurses. Learning from our past, we can enter a third wave of efforts, focused more on genomics integration. Concerted and strategic approaches by nurses in all domains of practice are needed to accelerate the creation and adoption of new genomic knowledge into education and practice. Preparing nurses to lead and practice at the forefront of innovation can optimize health outcomes. Noting how ideas, interests, institutions, and external factors in other countries generated different circumstances for nurses [30], leveraging nursing as a global profession can help create new strategies to advance genomics-informed nursing. We recommend three nurse-led strategies to rapidly respond to the anticipated professional practice changes arising from new evidence (see Table 1). (1) Establish collaboration across domains of practice to identify early signals of practice-changing research and reset priorities of leaders, faculty, professional associations, and regulators; (2) Create strategies to develop strong nursing leadership to facilitate policy and workforce development and implementation efforts toward health systems transformation; and (3) Develop a nursing research framework to produce evidence that can guide practice changes and avoid duplication of similar studies with the emergence of new bodies of evidence (see Figure 1).

### 4.1. Collaborate Across the Domains of Nursing Practice, Professions, and Sectors to Reset Priorities in Response to Emerging Evidence

Workforce development and health system redesign require awareness of how competing priorities, technology, evidence, and policy influence people to identify and set priorities [69,70,71,72]. The comparative policy analysis that used the 3i+E framework [30] explains how interests, ideas, institutions, and external factors impact the resetting of priorities of healthcare profession and institutions, leading to crucial infrastructure development. In the US and UK, early adopters who recognized the importance of genomics for nursing practice guided several initiatives to drive action and assist nursing organizations in genomics integration. At the time, initiatives were enhanced by solid buy-in and support from nurse leaders in academia, education, government and professional organizations who responded to the new evidence in genomics and advanced education, research, and nursing policy to support genomics integration [30]. In Canada, we learned that in the oncology setting, patient need, safety, and public demand were key factors influencing nurses’ views on the relevance and urgency of engaging with genomics [20,21]. However, there were a few formal mechanisms to relay early change signals to groups such as faculty, regulators, professional associations, or structures to facilitate changing priorities and infrastructure development. Mechanisms were also lacking to support interprofessional and cross-sector collaboration to clarify unique and overlapping practices, prevent or remove silos in genomics services, or optimize nurses’ contributions. Establishing networks to identify, act upon and sustain actions is crucial to mobilizing nurses from the five domains of practice for concerted action.

As such, networks are needed to ensure leaders within various institutions are aware of practice-changing research and the need to reset priorities. Leveraging existing and new collaboration networks across domains of nursing and sectors can create responsive systems capable of concerting action and preventing siloed initiatives. Regular and purposeful dialogue between nurses from the five domains of practice and professions can expand understanding of how emerging evidence places new demands on nursing and patient care and impacts interprofessional collaboration. Nurses can then ascertain the tipping point when resetting priorities is warranted and determine the consequences of action or inaction on nurses’ future as integral members of interprofessional teams.

Progress will be slow if every profession, province, and country develops separate resources, policies, and curricula. Therefore, sharing resources is key. The Global Genomics Nurses Alliance (G2NA) developed assessment and implementation tools informed by implementation science [62,63] that include concerted strategies across domains of nursing and organizations. The CNG developed a toolkit [73] to support education and genomic literacy. A global consortium developed the ACCESS Framework which offers a standardized, systematic guide to practices aimed at closing disparities in genomics healthcare [74]. Other countries can freely adopt and adapt these resources to reduce duplication and promote resource sharing to build nurses’ confidence and capacity in delivering genomics-informed healthcare. A harmonized approach can accelerate genomics integration.

### 4.2. Education in Leadership, Policy and Practice for Rapid Scale-Up of Workforce and Health System Transformation

The healthcare workforce must expand their genomic capacity to ensure safe and equitable access to genomic services [10,24,75,76]. The limited clinical adoption of genomics in nursing is structural, modifiable, and amenable to leadership strategies [28,44,45]. Focused leadership is critical to mobilize over 450,000 Canadian nurses [77] and over 29 million nurses worldwide [78]. Competencies, standards of practice, position statements, and education frameworks provide direction, articulate boundaries and ensure nurses are knowledgeable collaborators in education, research, administration, policy, and direct care [30]. With the introduction of any new practice or knowledge, policy infrastructure developed by regulators, employers, patients, professional associations and accrediting bodies is needed to articulate roles, responsibilities, and ensure patient safety [43,79,80]. For nurse leaders to meaningfully engage in policy and implementation efforts that enhance genomics-informed healthcare, genomics must be recognized as a complex science with unique implementation challenges requiring cross-sector and interprofessional skills [23,28]. Nurse leaders must be educated and mentored as policy advocates with skills to develop and implement policies, clinical strategies, and pathways based on emerging evidence. These skills include advocating for funding to drive policy initiatives forward and being involved in provincial and national committees to ensure nursing perspectives are considered in policy development [22,23,66].

We recommend that an initial phase of workforce development in response to substantial shifts in evidence prioritize leadership education through a well-defined plan and financial resources. A national system where nursing schools, regulators, and professional associations have structures to support leadership development for scale and spread of genomic knowledge can strengthen nursing. With substantial shifts in evidence in genomics, undergraduate and graduate-level leadership courses must quickly develop curricula and case studies to prepare leaders [28]. Teaching leaders about implementation frameworks can guide the rapid scale-up of the workforce for implementation and evaluation of healthcare strategies [63,64,65,81,82,83]. Education and ongoing support for champions, including Advanced Practice Nurses in genomics can facilitate the implementation of genomics into clinical care [18,45,84].

### 4.3. Create a Research Framework That Generates Evidence to Guide Nursing Practice

Research on educational, leadership, practice and policy interventions is required to ensure nurses in all five domains of practice are adequately informed to deliver safe, equitable, and sustainable genomics-informed care. Research on questions relevant to the discipline of nursing is urgently needed. A strategic research plan for nursing-led genomics studies focused on understanding, developing, testing, intervening and implementing solutions to patients’ health challenges can guide knowledge production. In addition, this plan should include implementation science [82,83], align with the complex needs of nursing practice and address the barriers and facilitators to the integration of research findings into practice. As a large and global profession, nursing must quickly mobilize researchers to conduct interventional studies to generate knowledge for practice. Genomic scoping reviews show gaps in intervention studies, with most of the literature focused on healthcare provider topics such as knowledge and perceptions [18,31,56]. With few intervention or outcome studies to show if actions are successful, nurses remain under-supported for evidence-based practice.

We recommend establishing a research and knowledge mobilization strategy that anticipates shifts in evidence and ensures nurse researchers and funding bodies are poised to generate evidence to support practice improvements. Research can showcase nurses’ contributions to patient health outcomes and position nurses as valuable collaborators and leaders [85,86]. Embedding nurse researchers into clinical practice settings can generate interprofessional research questions and advance genomics-informed health care in learning health systems [60]. Collaboration across the domains of practice can identify clinically relevant research questions crucial to producing evidence for practice.

## 5. The Promise of Canadian Nursing and Genomics 3.0

The promising outcomes from implementing these three recommendations were demonstrated through a case study analysis [32], the development of a position statement, and a special interest group for the Canadian Association of Nurses in Oncology (CANO/ACIO) [87]. Engaging Canadian nurse leaders across educational institutions, regulatory bodies, and professional associations led to the start of infrastructure needed to accelerate and harmonize efforts for integrating genomics into nursing. For example, when Canadian leaders across jurisdictions and domains of practice were given the opportunity to learn about genomics, they worked together to develop strategies to support evolving genomics-informed professional practices. Existing frameworks such as the Assessment of Strategic Integration of Genomics Across Nursing (ASIGN) Maturity Matrix [63] and the G2NA Roadmap [62] were used to structure the events. Canadian nurse leaders agreed upon four unified strategies for genomics-informed nursing and the development of a toolkit to support genomic literacy [73]. A similar approach is being used to create a position statement for CANO/ACIO [87]. Co-creating a pan-Canadian strategy for workforce development in education, policy, research, and practice is a way to harmonize knowledge mobilization and enable healthcare innovation [32].

When equipped with genomic literacy, effective policy, and leadership, oncology nurses can provide patient education, initiate appropriate referrals to specialist services, participate in strategies to reduce service wait times, support new care pathways, and implement evolving models of care [88,89,90]. Nurses can help remove structural barriers to ensure populations have equitable opportunities to benefit from genomics-informed care [31], including pharmacogenomics and genetic testing access [91]. Genomically competent nurses are well-positioned to provide genomics-informed care across the continuum of cancer services, improve access, and optimize the benefits of genetic testing. Nurses require education to build competencies for the genomic era [68].

## 6. Conclusions

The nursing profession’s response to genomics reveals opportunities to improve and harmonize support for professional practice to ensure nurses are prepared to meet patient care needs, fully participate in interprofessional care, and lead health system transformation across jurisdictions. A coordinated response to practice-changing research can galvanize national and global efforts to support the scale and spread of evidence to improve nursing practice and patient health. Addressing the three recommendations can support professional practice and position nurses as leaders and collaborators in safe, ethical, and equitable genomics-informed care. Implementing all three recommendations can lead to a concerted approach among nursing organizations, education and practice institutions in promoting and stabilize the genomics nursing workforce. Genomics is a rapidly advancing science with a broad impact across the healthcare continuum, and in particular in cancer care. Exploring the structures that support workforce development and health system transformation in response to scientific advancements in genomics can help nurses improve patient health outcomes while also providing a focus for future evidence-based initiatives.

## Figures and Tables

**Table 1:** Recommendations and Key Strategies for Action.

Nursing and Genomics 3.0	Key Strategies for Action
Establish collaboration across domains of practice to identify early signals of practice-changing research and reset priorities of leaders, faculty, professional associations, and regulators	Establish formal mechanisms to ensure leaders within various institutions are aware of practice-changing research and the need to reset priorities. Leverage existing and new collaborative networks to concert action, prevent siloed initiatives and share resources. Regular and purposeful dialogue between nurses from the five domains of practice and interprofessional teams can expand understanding of how emerging evidence places new demands on nursing and patient care, and impacts interprofessional collaboration.
Create strategies to develop strong nursing leadership to facilitate policy and workforce development and implementation efforts toward health systems transformation	Established structures within nursing schools, regulators and professional associations to quickly develop curricula and case studies to prepare leaders to respond to substantial shifts in evidence. Teach leaders about implementation frameworks in general and that were developed specifically for new emerging evidence.
Develop a nursing research framework to produce evidence to guide practice changes and avoid duplication of similar studies with the emergence of new bodies of evidence.	Create a strategic research and knowledge mobilization plan for nursing-led studies that focuses on understanding, developing, testing, intervening and implementing solutions to patients’ health challenges. Quickly draw on the large and global nursing profession to mobilize researchers to conduct interventional studies to generate knowledge for practice.

## Data Availability

Information and data supporting this paper were obtained from previously published articles. The reference list provides information to access these articles.
